# Dynamics of maternal gene expression in *Rhodnius prolixus*

**DOI:** 10.1038/s41598-022-09874-7

**Published:** 2022-04-20

**Authors:** Agustina Pascual, Rolando Rivera-Pomar

**Affiliations:** 1 Centro de BioInvestigaciones (CeBio-CICBA), Universidad Nacional del Noroeste de la Provincia de Buenos Aires (UNNOBA), Avenida Presidente Frondizi 2650, 2700 Pergamino, Buenos Aires, Argentina; 2 Centro de Investigaciones y Transferencias del Noroeste de la Provincia de Buenos Aires (CITNOBA-CONICET), UNNOBA-UNSAdA, Consejo Nacional de Investigaciones Científicas y Técnicas (CONICET), Buenos Aires, Argentina; 3 Centro Regional de Estudios Genómicos, Facultad de Ciencias Exactas, Universidad Nacional de la Plata, Bvd 120 y 62, 1900 La Plata, Buenos Aires, Argentina

**Keywords:** Developmental biology, Genetics

## Abstract

The study of developmental processes in *Rhodnius prolixus* has recently advanced with the sequencing of the genome. In this work, we analyze the maternal gene expression driving oogenesis and early embryogenesis in *R. prolixus*. We examined the transcriptional profile of mRNAs to establish the genes expressed across the ovary, unfertilized eggs and different embryonic stages of *R. prolixus* until the formation of the germ band anlage (0, 12, 24, and 48 h post egg laying). We identified 81 putative maternal and ovary-related genes and validated their expression by qRT-PCR. We validate the function of the ortholog gene *Bicaudal-D* (*Rp-BicD*) by in situ hybridization and parental RNAi. Consistent with a role in oogenesis and early development of *R. prolixus*, we show that lack of *Rp-BicD* does not significantly affect oogenesis but impairs the formation of the blastoderm. Based on our findings, we propose three times of action for maternal genes during oogenesis and embryogenesis in *R. prolixus*.

## Introduction

During insect embryogenesis, a sequential series of dynamic processes that include cell division, growth and fate specification take place to establish the necessary components to give rise to a complete organism, playing a fundamental role to support the developmental process of the whole life cycle^1–3^.

There are three modes of insect embryogenesis: long, intermediate, and short germ embryogenesis^4^. Long germ embryogenesis is defined by the simultaneous establishment of all segmental fates at the blastoderm stage. This is a derived mode of embryogenesis, found in scattered species among the Holometabola, such as *Drosophila melanogaster*. These insects have polytrophic meroistic ovaries. In short or intermediate germ insects only the most anterior segments are specified before gastrulation, while the more posterior segments are generated and patterned progressively from a posterior region called the growth zone. This represents an ancestral type of insect embryogenesis, described in insect models such *Oncopeltus fasciatus*, *Rhodnius prolixus*, *Bombyx mori, Tribolium castaneum*. It corresponds to insects with telotrophic or panoistic ovaries^4–8^. A common feature across the different modes of embryogenesis is the loading of maternal mRNA transcripts and proteins in the egg during oogenesis^9^.

In the last 20 years the rise of new models for comparative insect development provided a framework to understand the genetic basis of development and evolution^10–17^. The different mechanisms of insect embryogenesis are determined by specific spatiotemporal gene expression patterns derived from common genetic programs, suggesting that the mechanisms are much more conserved than the diversity of germ types might suggest^4,18–22^. In addition, a detailed study of cell flow during germ band extension and the fate map of *T. castaneum* embryo led to the idea that short and long germ bands share many more common features than thought^23^. In the last decade the expanse of genomics and transcriptomic analysis provided an insight of the transcriptional basis of the embryonic development in non-model insect species^24^. However, the complete repertory of genes involved in oogenesis and early embryogenesis has been reported in detail only in *D. melanogaster*^25–29^ and *T. castaneum*^15,30,31^, remaining an open question in other model organisms.

The blood-feeding insect *Rhodnius prolixus* is one vector of *Trypanosoma cruzi*, the etiologic agent of Chagas disease^32,33^. In addition to its medical interest, it has been a classical model for physiology and biochemistry^34–37^. The embryonic development of *R. prolixus* has been described from fertilization to hatching^7^, and the process of oogenesis studied in detail^38–44^. The genome was recently sequenced^45^ and since then, *R. prolixus* is an emerging model for developmental biology^46–50^. Several transcriptome analyses were reported, focusing in the gene expression of the follicular epithelium, the early previtellogenic stage of oogenesis; as well, in the impact of the nutritional state on regulatory pathways associated with reproductive performance^51–53^. Very recently, a thorough study on previtellogenic ovaries and unfertilized eggs discovered a large number of unannotated genes in the *R. prolixus* genome and unveiled a large set of maternal genes^54^. With all this knowledge in place, we have moved forward to the understanding of the genetic and molecular mechanisms driving oogenesis and the maternal contribution to embryo patterning in *R. prolixus*. Here, we present a transcriptome profiling approach to identify the genetic basis underlying oogenesis and early embryogenesis of *R. prolixus* until the onset of gastrulation, with a focus on genes related to embryonic patterning and egg formation. We provide novel insight into the molecular basis of early embryo formation and show the dynamic of mRNA expression during early embryo development in *R. prolixus*. Our study provides maternal and early embryonic transcriptomes of this hemimetabolous insect. We present a comprehensive qualitative data about genes related to segmentation, dorsal ventral axis and oogenesis, validate gene expression by qRT-PCR and show the phenotype of *Bicaudal D* (*BicD*) homolog likely related to early steps of the maternal cascade that leads to patterning.

## Materials and methods

### Insect rearing

A colony of *R. prolixus* was maintained in our laboratory in a 12:12 h light/dark period at 28 °C and 80% relative humidity in controlled environment incubators. In these conditions, embryogenesis takes 14 ± 1 days. Insects were regularly fed on chickens, which were housed, cared, fed and handled in accordance with resolution 1047/2005 (Consejo Nacional de Investigaciones Científicas y Técnicas, CONICET) regarding the bioethical framework for biomedical research with laboratory, farm, and nature collected/wild animals. This framework is in accordance with international standard procedures. Biosecurity considerations agree with CONICET resolution 1619/2008, which is in accordance with the WHO Biosecurity Handbook (ISBN 92 4 354 6503).

### Sample collection, RNA isolation and sequencing

Adult mated insects 6th days after the feeding regimen were used to collect fertilized eggs at specific points in developmental time—0 (zygote), 12 (blastoderm), 24 (cellular blastoderm) and 48 (onset of germ band formation) hours post egg laying (*hPL*). Virgin female adults were used to collect unfertilized eggs, which were immediately frozen and stored in liquid nitrogen. At the same time, female ovaries were dissected in the vitellogenic stage and placed in a cryotube containing Trizol (Invitrogen), flash frozen and stored in liquid nitrogen until use.

For the transcriptome profiling, RNA was extracted from 150 embryos for each developmental time and 30 vitellogenic ovaries. For qRT-PCR analysis, independent experiments were carried out using 75 embryos from each specific time and 10 vitellogenic ovaries. Total RNA was isolated using Trizol (Invitrogen) as recommended by the manufacturer. RNA integrity was determined by agarose electrophoresis and concentration measured using Qubit RNA Assay Kit in a Qubit 2.0 Fluorometer (Life Technologies, Invitrogen). cDNA libraries were synthetized from 1 µg of total RNA and sequenced using a HiSeq-3000 platform (Illumina) to obtain the 50 base pairs (bp) (single-end) or 150 bp (paired-end) reads. The RNA-seq data has been submitted to the NCBI SRA database, available under accession code PRJNA694974.

### Quality control, alignment and transcriptome assembly

Raw data were processed with FASTX-toolkit software (http://hannonlab.cshl.edu/fastx_toolkit/), to remove adapter sequences, reads with unknown bases and reads with quality scores lower than Q30, showed by the FastQC report (http://www.bioinformatics.babraham.ac.uk/projects/fastqc/). To avoid contaminants, the presence of adaptor sequences was ruled out using BLASTn^55^ and the UniVec database (ftp://ftp.ncbi.nlm.nih.gov/pub/UniVec/) from NCBI. Additionally, to remove rRNA sequences the SILVA database was used^56^. The remaining reads were defined as clean reads and used for subsequent bioinformatics analyses. TopHat2^57^ was used to map clean reads to the ab initio annotations of *R. prolixus*, genome dataset version RproC3.3^45,58^. The mapping statistics by the RNA-seq reads were calculated by using bam_stat.py implemented in the RSeQC package^59^ and the advanced statistics of coverage analysis were performed by the Qualimap application^60^. After TopHat alignment, transcripts were assembled using Cufflinks^61,62^. Assembly quality was assessed for each assembly using BUSCO analysis^63^, with the reference gene set of arthropods (2676 proteins) with default parameters. Fasta Statistics was used to display summary statistics from each transcriptome generated^64^. The eggNOG 5.0 database was used for functional annotation of the transcripts with common denominators or functional categories (i.e., derived from the original COG categories). Also, predicted protein-coding transcripts^65^ were functionally annotated. For each protein sequence protein signatures were assigned, using InterProScan search Version 5.0.0^66^, through the PfamA and SuperFamily databases. Proteins annotated by signatures were assigned into GO (Gene Ontology) categories, including biological processes (BP), molecular functions (MF) and cellular components (CC). To statistically analyze GO-term enrichment, topGO package^67^ was implemented, using Fisher’s exact test and the false discovery rate (FDR) adjusted method. A q-value smaller than 0.05 were considered as significant. The reference set of gene-to-GO mappings was available from VectorBase (https://www.vectorbase.org/).

### Oogenesis and early embryogenesis gene identification

Gene identification was performed using local BLAST^55^ on the six transcriptome assemblies. The BLAST algorithm used was BLASTx. The search was limited to 84 protein sequences derived from FlyBase (Version FB2020_03, https://flybase.org/), comprising genes related to oogenesis and early embryogenesis, with an e-value threshold of 10^−5^. Transcript with blast hit to *Drosophila* were then manually checked by BLASTx against all Arthropoda protein sequences (NCBI non-redundant protein (nr) database, assessed January 2018) to confirm sequence identity. BLAST results were classified into the known *D. melanogaster* developmental process. For the maternal gene search, a database was generated from different resources containing 10,277 specific protein sequences (Additional file 1)^15,25–29^.

### Quantitative real-time PCR

Total RNA was isolated using Trizol reagent (Invitrogen) and treated with DNAse (QIAGEN). cDNA was synthesized using SuperScript™ VILO™ MasterMix kit (Invitrogen) following the manufacturer’s instructions. PCR was performed in technical triplicates (3 wells/cDNA sample), in a 10 μl final volume as follows: (i) 95 °C for 10 min; (ii) 95 °C for 15 s; (iii) 55 °C for 30 s; (iv) 72 °C for 45 s; (v) steps (ii) to (iv) for 35 cycles. Gene expression level was quantified using SsoAdvanced Universal SYBR Green Supermix (Bio-Rad) in an Applied Biosystems 7500 Real-Time PCR System (Thermo Fisher Scientific). A control without a template was included in all batches and *α-tubulin* was used as reference, after a screen of several housekeeping gene candidates, as it provided consistent results on the embryonic stages analyzed. All primer pairs (Additional file 2) were tested for dimerization, efficiency, and amplification of a single product. The Ct value was averaged for the technical triplicate experiments and subtracted from the average Ct of the reference gene, to yield the expression difference (dCt) for each biological replicate. The results were analyzed according to^68^. To test whether the expression of a given gene was significantly different across developmental times, a one-way ANOVA was carried out followed by post-hoc test using GraphPad Prism v6.0 software (GraphPad Software, CA, USA, www.graphpad.com).

### In situ Hybridization and Parental RNAi

DNA templates used to synthesize in situ hybridization probes were obtained by PCR using oligonucleotides carrying T7 promoter sequences at the 5'-end. The templates, bearing T7 promoter either in the sense or antisense direction, were in vitro transcribed using DIG RNA labeling kit (Roche) to produce sense and antisense probes. In situ hybridization was performed as described in Pascual et al.^50^. Female ovaries (n = 11) were dissected during the vitellogenic stage and eggs (n = 40) collected at specific developmental times.

For parental RNAi, dsRNA was produced by simultaneous transcription with T7 RNA polymerase (New England Biolabs) on templates containing T7 promoter sequences at both ends. The amplicons were sequenced to confirm identity (Macrogen Inc.). dsRNA^*BicD*^ was quantitated by fluorescence and injected into virgin females as described in Lavore et al.^69^. Two days after injection, the females were fed to induce oogenesis and mated. After mating, eggs were collected and ovaries fixed as described^50^. Phenotypic analysis of the ovaries was always performed at the vitellogenic stage. A negative control was performed injecting virgin females with dsRNA corresponding to the β-lactamase gene (dsRNA^β-lac^) of *E. coli*^69^. Oligonucleotides used in this study are listed in Additional file 2.

## Results and discussion

### Assembling the ovarian and early embryonic transcriptomes of *R. prolixus*: characterization and completeness analysis

The RNA-seq output comprises six *R. prolixus* samples that cover late oogenesis to the beginning of germ band extension (48 *hPL*). Statistics on the sequencing and mapping are reported in Table 1A. According to completeness analysis, the coverage metrics obtained indicate that the assembled transcriptomes are sufficient for a meaningful analysis (Table 1B). As the genomic reference has a reasonable number of positions that are not called, transcriptomes assembled by mapping genomic predictions (ab initio) were used for subsequent analyses. In this respect, a review of zygotic genes^14^ and of the regulatory pathways involved in egg production^52^ has been reported based on these genomic annotations with a robust gene identification.

**Table 1:** Summary of the RNA-seq metrics from *R. prolixus* transcriptomes from vitellogenic ovaries, unfertilized and 0 to 48 *hPL* eggs. **A**: Raw Reads: the original sequencing reads counts; Clean Reads: number of reads after filtering; % read mapping rate: percentages of the overall mapping rate using the annotated genome as reference, % GC: percentages of G and C in total bases. **B**: Transcriptome assembly’s statistics, Number, number of the total of reconstructed transcripts; Mean length: the mean length in base pairs; N50: is the size of the transcript which, along with the larger transcripts, contain half of sequence of the reference; GC: percentages of G and C in total bases; BUSCO: percentages of the transcriptome completeness by BUSCO analysis results.

Transcriptome	Raw reads	Clean reads	% read mapping rate	GC (%)	
*Vitellogenic ovary*	R1 187,726,140	R1 139,828,293	27.00	37.52	
	R2 186,676,153	R2 139,828,293			
*Unfertilized*	R1 186,676,153	R1 126,233,152	40.80	48.69	
	R2 184,269,740	R2 126,233,152			
*0 hPL*	R1 127,066,601	R1 96,455,551	14.80	41.9	
	R2 196,653,388	R2 96,455,551			
*12 hPL*	80,970,506	80,924,542	49.00	39.41	
*24 hPL*	34,236,005	34,178,892	39.40	39.47	
*48 hPL*	48,825,374	48,790,775	54.10	36.90	

Transcriptome	Number	Mean length	N50 (nt)	GC (%)	BUSCO (%)
*Vitellogenic ovary*	12.266	997	1.254	38.1	88.1
*Unfertilized*	11.141	1.070	1.360	39	88.9
*0 hPL*	10.655	1.080	1.338	38.8	87.4
*12 hPL*	11.285	1.015	1.344	38.7	88.7
*24 hPL*	11.437	909	1.187	38.7	88.0
*48 hPL*	12.239	1.035	1.376	38.8	90.1

**Figure 1:**
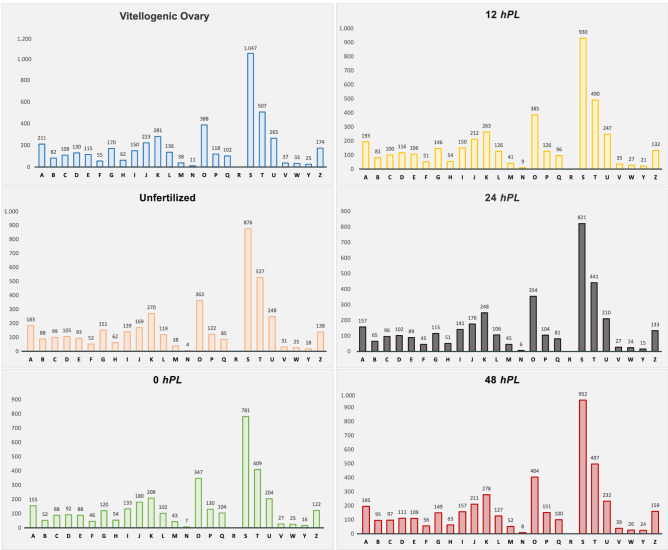
Classification of eggNOG annotations in the *R. prolixus* transcriptomes. The capital letters on the X-axis represent different eggNOG categories. Y-axis shows the number of transcripts in each eggNOG category. A: “RNA processing and modification”, B: “Chromatin structure and dynamics”, C: “Energy production and conversion”, D: “Cell cycle control, cell division, chromosome partitioning”, E: “Amino acid transport and metabolism”, F: “Nucleotide transport and metabolism”, G: “Carbohydrate transport and metabolism”, H: “Coenzyme transport and metabolism”, I: “Lipid transport and metabolism”, J: “Translation, ribosomal structure and biogenesis”, K: “Transcription”, L: “Replication, recombination and repair”, O: “Post-translational modification, protein turnover, and chaperones”, P: “Inorganic ion transport and metabolism”, Q: “Secondary metabolites biosynthesis, transport, and catabolism”, R: “General function prediction only”, S: “Function unknown”, T: “Signal transduction mechanisms”, U: “Intracellular trafficking, secretion, and vesicular transport”, V: “Defense mechanisms”, W: “Extracellular structures”, Y: “Nuclear structure”, Z: “Cytoskeleton”.

In order to conduct a transcriptome-composition representation analysis, eggNOG analysis was performed. A total of 25 eggNOG categories were detected (Fig. 1 and Additional file 3), in which the category “function unknown” was dominant followed by “Signal transduction mechanisms” and “Post-translational modification, protein turnover, and chaperones” in all the analyzed developmental times. To obtain information of the predicted proteins, InterproScan searches were performed to identify functional domains, repeats, sites and protein families conserved in the protein-coding transcripts (Table 2 and Additional file 4). For all of the characterized transcripts, statistically over-represented GO terms were identified using the FDR adjusted relative to a reference set of 11,947 genes. These statistically highlighted GO terms were summarized to generic GO categories for each developmental time studied (Table 2 and Additional file 5). GO analysis showed that mainly metabolic processes were enriched during embryo development, such as cellular macromolecule metabolic process (GO: 0044260), nucleobase-containing compound metabolic process (GO: 0006139), organonitrogen compound biosynthetic process (GO: 1901566), gene expression (GO: 0010467). This enrichment is in agreement with the requirements of the embryo during the transitions between the different embryonic stages with rapidly changing of the anabolic and catabolic demands^70,71^.

**Table 2:**
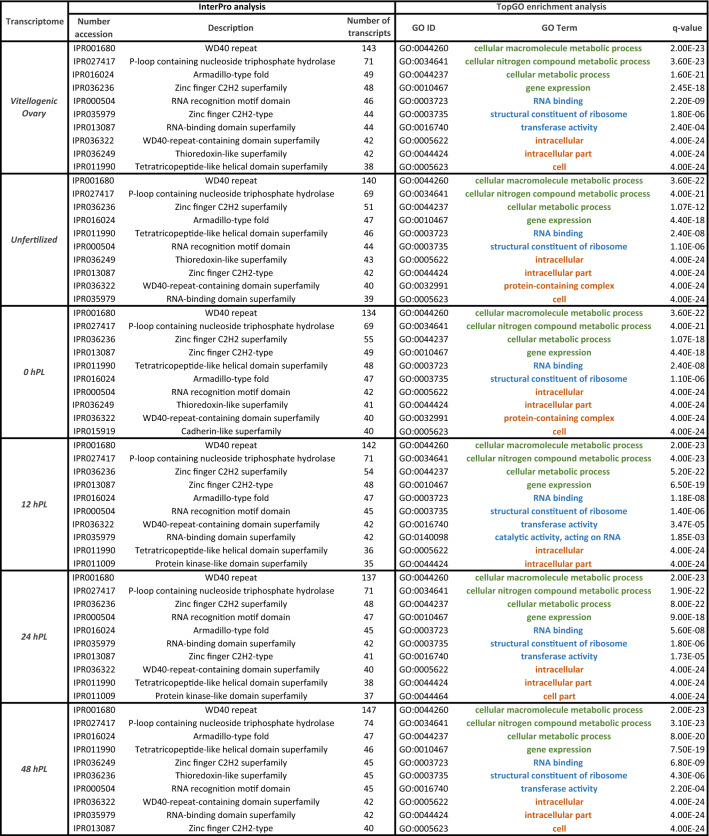
Summary of the functional annotation. Top 10 Interpro domains and GO terms enriched in each transcriptome analyzed. TopGO results were shown in different colors according to the three sub-ontologies: green “Biological Process”, blue “Molecular Functions” and orange “Cellular Component”.

**Figure 2:**
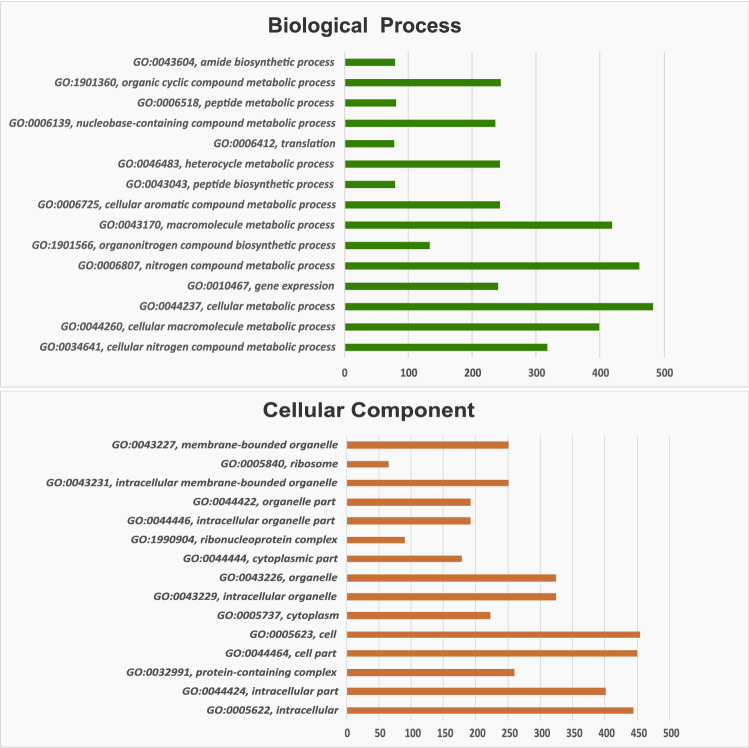
Bar graph of Gene Ontology (GO) enrichment analysis of the common transcripts across the six developmental stages. Upper: GO category "Biological process", lower: "Cellular component". X-axis: number of transcripts involved in the distinct GO terms. Y-axis: description of GO terms with the GO ID.

A total of 1192 annotated transcripts common to all developmental stages studied were further examined to determine GO enrichment. The represented GO terms (Fig. 2 and Additional file 6) were categorized in two main groups: cellular components and biological processes. The main terms of cellular components are cell structures related to protein synthesis, while the terms of biological processes are related to lipid, carbohydrate, nucleic acid and protein metabolism, all significantly over-represented. As expected in the enrichment observed for each developmental time, these biological processes play key roles in the embryonic development of *R. prolixus*. Energy is supplied during embryogenesis by the breakdown of biomolecules stored in the yolk^72^. This, in turn, drives the anabolic pathways such as protein and nucleotide biosynthesis to meet the needs of the developing embryo^70^. These also agree with the increment of lipid, protein and carbohydrate biosynthesis reported during embryonic stages of *T. castaneum*, *Boophilus microplus* and *Aedes aegypti*^73–75^, and in fed females of *R. prolixus* and other triatomines^76–79^.

### Gene identification for developmental processes

In order to study genes involved in early embryogenesis and oogenesis, a list of 84 developmental genes (37 segmentation-related genes, 16 of the dorsal patterning pathway and 31 linked to oogenesis) related to these processes in *D. melanogaster* was used to identify ortholog sequences in the *R. prolixus* transcriptomes (Table 3A-C). The approach identified 81 expressed genes conserved in *R. prolixus*. We cannot rule out that the absence of a transcript is due to an incomplete transcriptome coverage rather than evolutionary divergence. It is plausible to consider that some genes are expressed at very low levels or in a small subset of cells in the developmental stages analyzed and that enrichment analysis may be necessary for their identification. The genes *gurken* (*grk*), *bicoid* (*bcd*), *oskar* (*osk*), were not included in the search because they have been reported absent in triatomines^14^. We observed in some cases, as we previously had^50^, that the reads that correspond to a single assembled transcript correspond to two different gene ID. Rather than corresponding to duplicated genes (paralogs), manual curation and assembly of the sequences revealed that both gene ID corresponded to a single transcriptional unit. Our work and the remarkable progress in the annotation of early genes by Coelho et al.^54^ suggest that the current version of the genome and the annotated gene IDs need to be revised or that the genome needs long read resequencing to fill gaps in the assembled sequence.

**Table 3:**
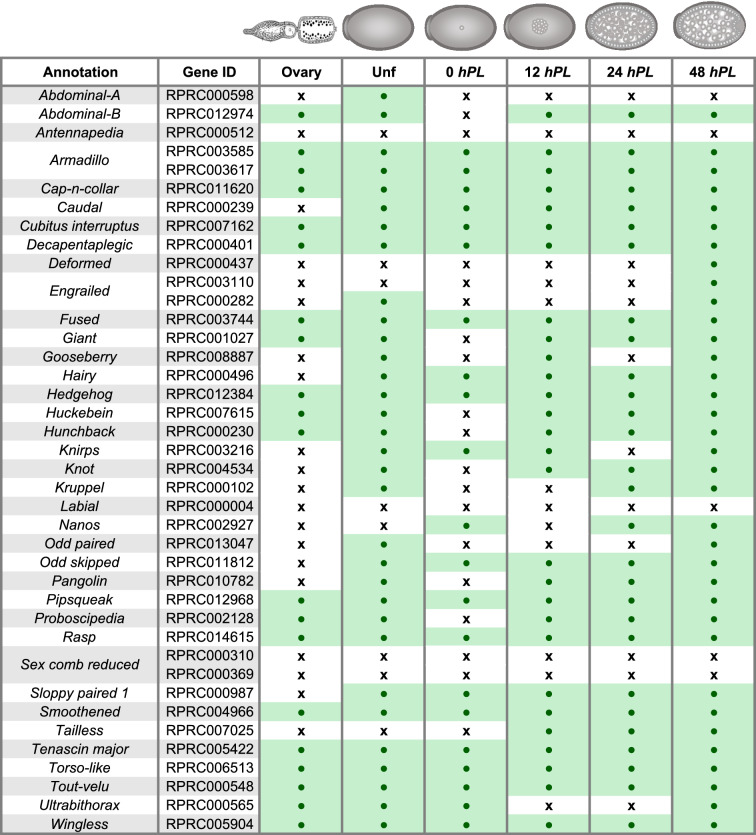

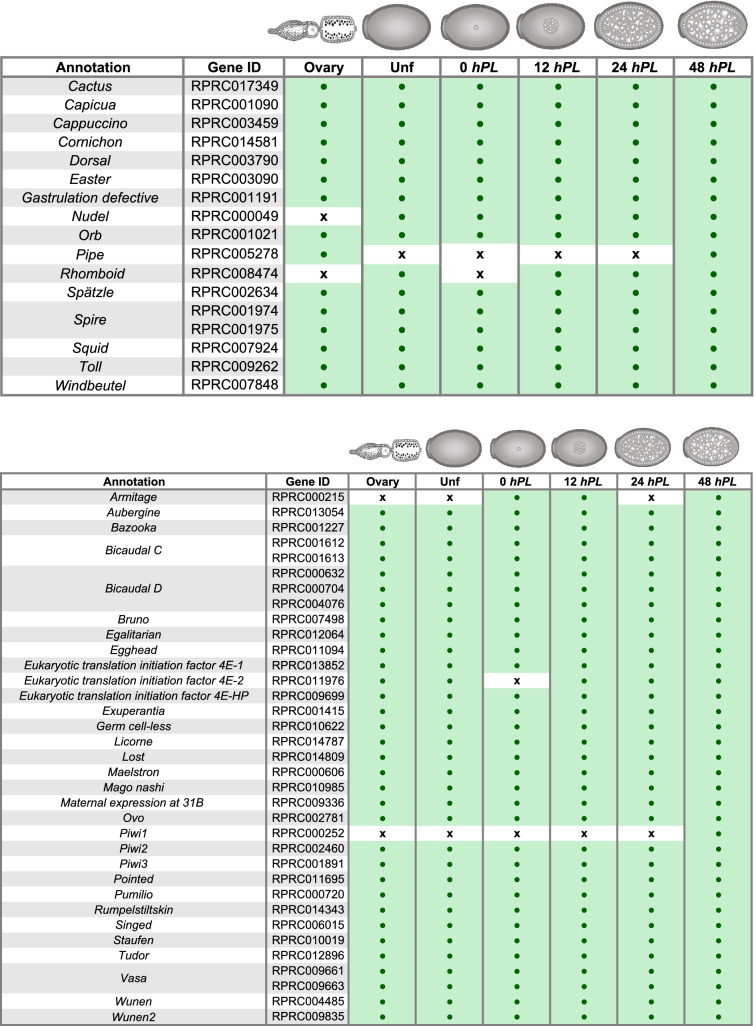
Developmental genes identified in the *R. prolixus* transcriptomes. Gene ID: VectorBase code (the official gene number in the RproC3 genome assembly); Annotations: protein name we are assigning. For each annotation, presence/absence was assessed by sequence similarity search. Green boxes indicate presence of expression; white boxes indicate absence of expression. **A:** Segmentation genes, **B:** Dorso-ventral genes, **C:** oogenesis, orthologues *piwi* genes were annotated as Brito, et al.^48^.

One would expect that genes related to oogenesis should not be expressed in embryonated eggs at gastrulation and germ band formation (48 *hPL*), as well as zygotic expression genes should not be expressed in unfertilized eggs. However, our analysis of different developmental stages revealed a number of genes expressed throughout early embryogenesis. Thus, all genes showed maternal expression. These results, although surprising, would agree with the role of the maternally active genome as director of most of early animal development^15,80–83^. In *D. melanogaster* and *T. castaneum* a high percentage of maternal transcripts are deposited during oogenesis, and so were detected (approximately 58–65%) in the course of the first hours of embryo development, while zygotic transcription was not detected^15,25,27,83^. These maternal mRNAs have been reported to have several functions, including the establishment of polarization gradients^84–86^, segregation of cell-fate determinants^87–95^, and targeting of protein synthesis to specialized organelles or cellular domains^96–100^.

**Figure 3:**
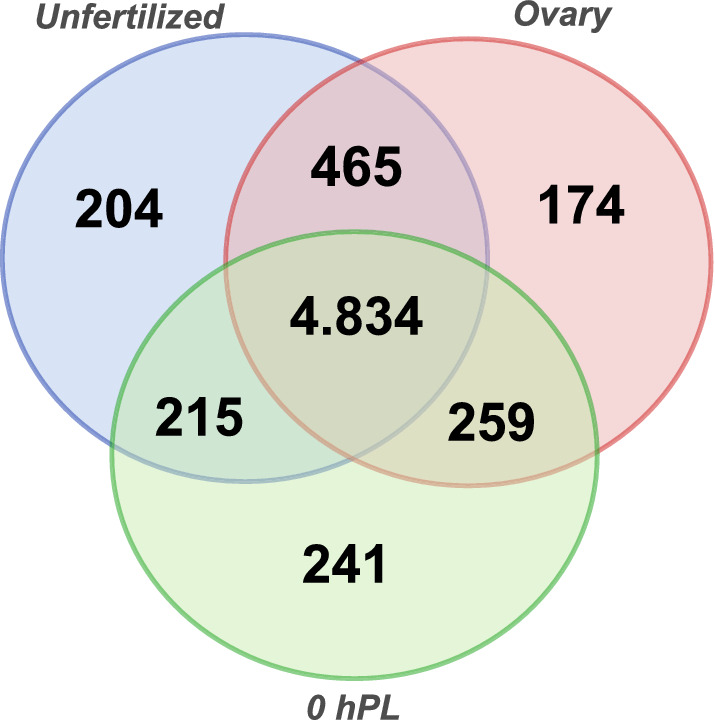
Venn diagram depicting the maternal transcripts across developmental stages. Comparison among the expressed transcripts of the Vitellogenic ovary, Unfertilized and 0 *hPL*, eggs and reference maternal genes.

To gain insight into this maternal contribution, ovary, unfertilized and 0 *hPL* transcriptomes were used to examine the expression of *R. prolixus* orthologues of genes reported maternal in *D. melanogaster.* The dataset was comprised of 10,277 sequences, of which the 54% had a *R. prolixus* ortholog to the *D. melanogaster* genes (Fig. 3 and Additional file 7). 40.7% were expressed during the three stages, oogenesis, unfertilized and 0 *hPL* eggs, 1.46% of the maternal genes had *R. prolixus* orthologs which were expressed only during oogenesis but not in deposited eggs, 1.72% had *R. prolixus* orthologs that were only maternally loaded into unfertilized eggs, and 2.03% only showed expression in the first hours of fertilized eggs. A similar analysis was reported with unfertilized eggs of *T. castaneum* with respect to *D. melanogaster* maternal genes*.* Here we identify in *R. prolixus* 306 maternal orthologs in addition to the ones reported in *T. castaneum*^15^. Out of 81 developmental genes investigated, eight were not reported to be maternal neither in *D. melanogaster* nor *T. castaneum* (Table 4). These eight genes were identified in the transcriptome of unfertilized eggs (*gooseberry*: RPRC008887, *odd paired*: RPRC013047, *odd skipped*: RPRC011812, *rhomboid*: RPRC008474, *sloppy paired*: RPRC000987), vitellogenic ovary (*pipe*: RPRC005278), or in both, vitellogenic ovary and unfertilized eggs (*windbeutel*: RPRC007848 and *proboscipedia*: RPRC002128). We also identified a subset of transcripts derived from the *Hox* cluster: *abdominal-A* (RPRC000598), *Abdominal-B* (RPRC012974), *Deformed* (RPRC000437), *proboscipedia* and *Ultrabithorax* (RPRC000565) (Table 3A, Table 4), suggesting maternal/zygotic transcription of the HOX genes in *R. prolixus*.

**Table 4:** Annotated developmental genes identified as maternal gene in *R. prolixus.* + : detected with maternal expression; − : not detected.

Annotation	Gene ID	Maternal expression in:		Annotation	Gene ID	Maternal expression in:	
		*Dm*	*Tc*			*Dm*	*Tc*
*Abdominal-A*	RPRC000598	+	+	*Licorne*	RPRC014787	+	−
*Abdominal-B*	RPRC012974	+	+	*Lost*	RPRC014809	+	−
*Armadillo*	RPRC003585	+	+	*Maelstron*	RPRC000606	+	+
	RPRC003617	+	+	*Mago nashi*	RPRC010985	+	−
*Armitage*	RPRC000215	+	+	*Maternal expression at 31B*	RPRC009336	+	+
*Aubergine*	RPRC013054	+	−	*Nanos*	RPRC002927	+	−
*Bazooka*	RPRC001227	+	+	*Nudel*	RPRC000049	+	−
*Bicaudal C*	RPRC001612	+	−	*Odd paired*	RPRC013047	−	−
	RPRC001613	+	−	*Odd skipped*	RPRC011812	−	−
*Bicaudal D*	RPRC000632	+	+	*Orb*	RPRC001021	+	+
	RPRC000704	+	+	*Ovo*	RPRC002781	+	−
	RPRC004076	+	+	*Pangolin*	RPRC010782	+	+
*Bruno*	RPRC007498	+	+	*Pipe*	RPRC005278	−	−
*Cactus*	RPRC017349	+	−	*Pipsqueak*	RPRC012968	+	−
*Capicua*	RPRC001090	+	+	*Piwi1*	RPRC000252	+	+
*Cap-n-collar*	RPRC011620	+	+	*Piwi2*	RPRC002460	+	+
*Cappuccino*	RPRC003459	+	+	*Piwi3*	RPRC001891	+	+
*Caudal*	RPRC000239	+	+	*Pointed*	RPRC011695	+	+
*Cornichon*	RPRC014581	+	−	*Proboscipedia*	RPRC002128	−	−
*Cubitus interruptus*	RPRC007162	+	+	*Pumilio*	RPRC000720	+	−
*Decapentaplegic*	RPRC000401	+	−	*Rasp*	RPRC014615	+	−
*Deformed*	RPRC000437	+	+	*Rhomboid*	RPRC008474	−	−
*Dorsal*	RPRC003790	+	+	*Rumpelstiltskin*	RPRC014343	+	+
*Easter*	RPRC003090	+	−	*Singed*	RPRC006015	+	−
*Egalitarian*	RPRC012064	+	+	*Sloppy paired 1*	RPRC000987	−	−
*Egghead*	RPRC011094	+	−	*Smoothened*	RPRC004966	+	−
*Eukaryotic translation initiation factor 4E-1*	RPRC013852	+	−	*Spätzle*	RPRC002634	+	+
*Eukaryotic translation initiation factor 4E-2*	RPRC011976	+	−	*Spire*	RPRC001974	+	+
*Eukaryotic translation initiation factor 4E-HP*	RPRC009699	+	−		RPRC001975	+	+
*Engrailed*	RPRC003110	+	+	*Squid*	RPRC007924	+	−
	RPRC000282	+	+	*Staufen*	RPRC010019	+	+
*Exuperantia*	RPRC001415	+	+	*Tailless*	RPRC007025	+	+
*Fused*	RPRC003744	+	−	*Tenascin major*	RPRC005422	+	−
*Gastrulation defective*	RPRC001191	+	−	*Toll*	RPRC009262	+	+
*Germ cell-less*	RPRC010622	+	+	*Torso−like*	RPRC006513	+	+
*Giant*	RPRC001027	+	+	*Tout−velu*	RPRC000548	+	+
*Gooseberry*	RPRC008887	−	−	*Tudor*	RPRC012896	+	−
*Hairy*	RPRC000496	+	+	*Ultrabithorax*	RPRC000565	+	+
*Hedgehog*	RPRC012384	+	−	*Vasa*	RPRC009661	+	+
*Huckebein*	RPRC007615	+	+		RPRC009663	+	+
*Hunchback*	RPRC000230	+	+	*Windbeutel*	RPRC007848	−	−
*Knirps*	RPRC003216	+	−	*Wingless*	RPRC005904	+	+
*Knot*	RPRC004534	+	−	*Wunen*	RPRC004485	+	+
*Kruppel*	RPRC000102	+	+	*Wunen2*	RPRC009835	+	−

Taken together, our data support the notion that maternal expression of developmental genes is widespread in *R. prolixus* and maintained (either by mRNA stability or zygotic transcription) during early developmental stages. The results agree with other gene specific studies that have reported maternal expression in the triatomine, during oogenesis and embryo development^46,48,50,69^. This compelling feature deserves further investigation, however it is currently limited by the inability to discriminate maternal and zygotic effects in *R. prolixus* by parental RNAi.

### Maternal gene expression validation

**Figure 4:**
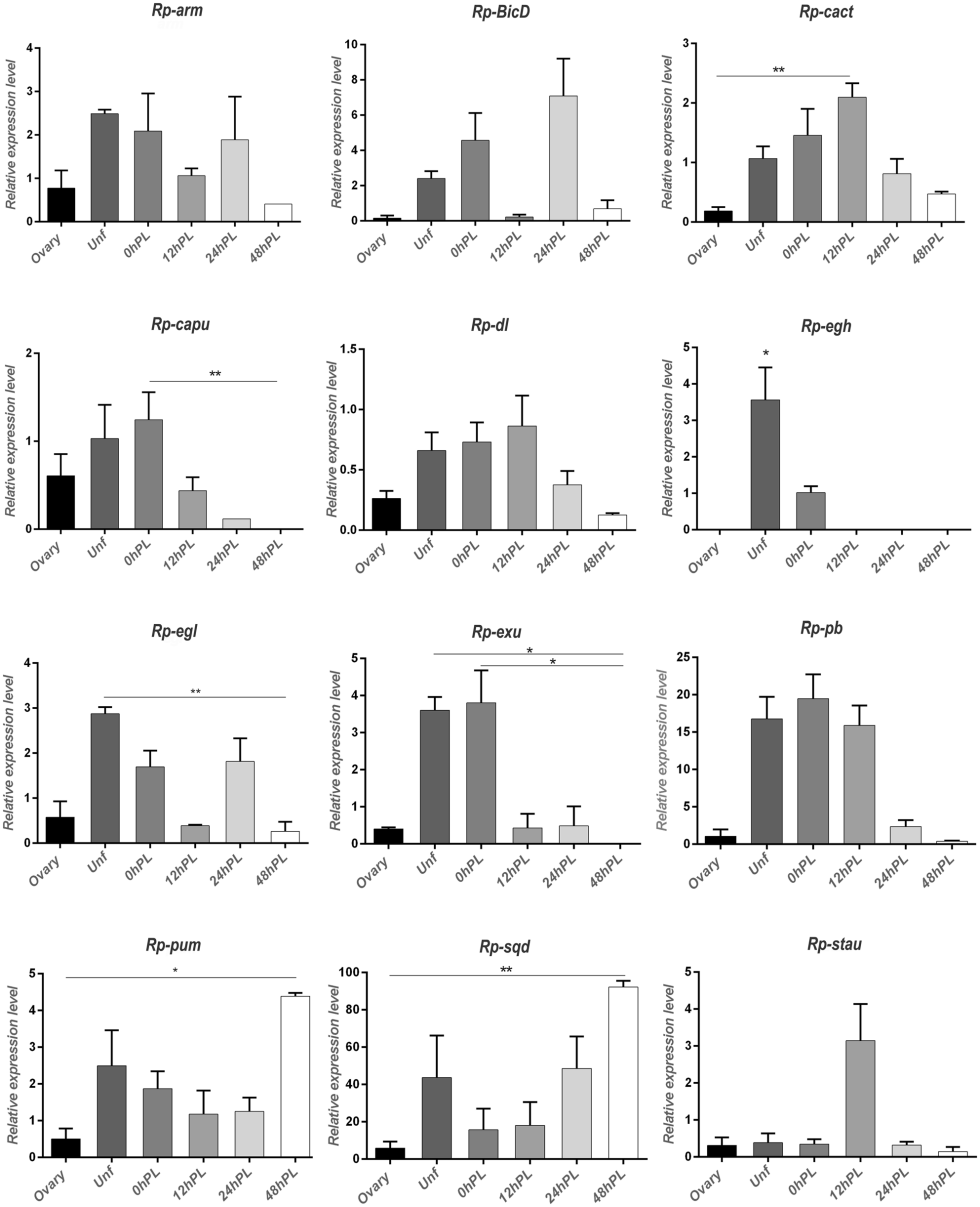
Real-time quantitative PCR of expression of candidate genes in the different stages. X axis: developmental times analyzed. Y axis: expression relative to the reference gene. Values are expressed as mean ± SEM of 3 independent experiments. *Rp-arm*: *Armadillo*; *Rp-BicD: Bicaudal D*; *Rp-cact: Cactus*; *Rp-capu: Cappuccino*; *Rp-dl: Dorsal*; *Rp-egh: Egghead*; *Rp-egl: Egalitarian*; *Rp-exu: Exuperantia*; *Rp-pb: Proboscipedia*; *Rp-pum: Pumilio*; *Rp-sqd: Squid*; *Rp-stau: Staufen*. Graphs were performed using GraphPad Prism 7. * < 0.1; ** < 0.05.

To corroborate the gene expression patterns revealed by the transcriptomic analyses, 12 genes out of 81 genes involved in oogenesis and early embryo development were chosen for qRT-PCR. The analysis was performed with three independent biological replicates different from those used for RNA-seq. The relative transcript levels over time are shown in Fig. 4. All of the selected genes showed transcript variation consistent with the results derived from the transcriptome analysis, indicating that our approach, although it was not intended to be quantitative, was valid for the identification of expressed genes. All the genes analyzed showed presence of the mRNA in unfertilized eggs, which confirms that they were maternally provided. The different expression level between the vitellogenic ovary and the unfertilized egg could be explained by the nature of the samples, which implied ovarioles depleted of choriogenic egg chambers. *Rp-sqd* showed higher relative transcript levels than the other genes of interest at the different analyzed times.

**Figure 5:**
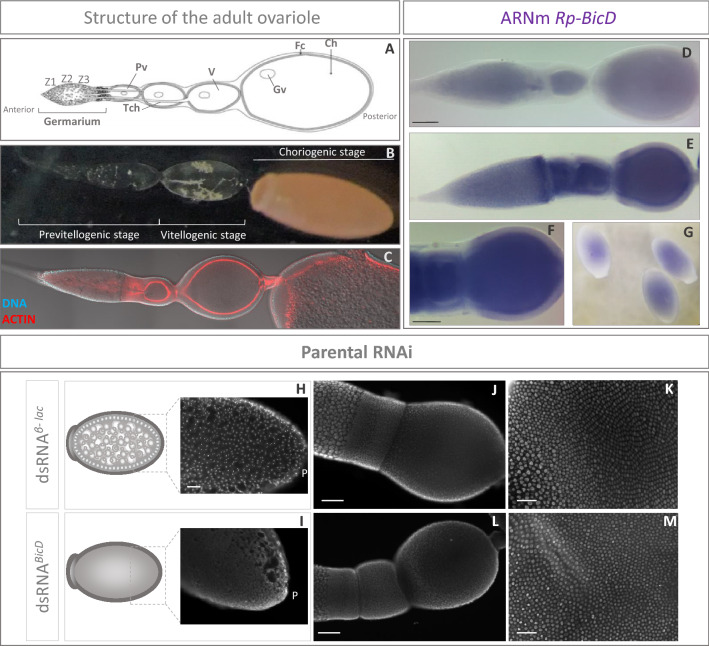
Silencing of *Rp-BicD* produces anembryonic eggs. (**A**) Schematic of the ovariole showing the germarium host mitotically active cells (i.e. nurse cells) in zone 1 (Z1), zone 2 (Z2) and zone 3 (Z3), and previtellogenic (Pv), vitellogenic (V) and choriogenic (Ch) oocytes. Each oocyte becomes encapsulated by follicle cells (Fc), and remains connected to the germarium through the trophic cords (Tch). Germinal vesicle (Gv). Schematic representation adapted from McLaughlin and Bratu^116^. (**B**) Structure of the ovariole showing the different stages that characterized oogenesis: previtellogenic, vitellogenic and choriogenic stage. (**C**) Ovariole of a control female showing the nuclei distribution by DAPI staining and the actin filaments by phalloidin staining^50,117^. (**D**) Detection of the sense probe by in situ hybridization assays in early stages of oogenesis. Scale Bar: 20 µm. (**E**) Detection of *Rp-BicD* transcript in the germarium and follicular cells of the ovariole with the antisense probe. Note the detection in Z3 of the germarium indicates *Rp-BicD* specific expression. Scale Bar: 20 µm. (**F**) Higher magnification of the developing oocytes. Notice the expression of *Rp-BicD* in the follicular cells. Scale Bar: 50 µm. (**G**) Detection of *Rp-BicD* transcript in unfertilized eggs by in situ hybridization. The arrowhead indicated the region of transcript accumulation. (**H**) Blastoderm embryo derived from control females. Note the nuclei distribution in the surface as revealed by DAPI staining. Scale bar: 100 µm. P: Posterior pole of the egg. (**I**) Egg from silenced (RNAi^*BicD*^) females. Note the lack of nuclei in the surface of the embryo. Scale bar: 100 µm. P: Posterior pole of the egg. The developmental stages corresponding to both, control (G) and silenced (H), represent the cellular blastoderm (24 *hPL*). (**J**) Ovariole of a control females showing the nuclei distribution by DAPI staining. Scale bar: 100 µm. (**K**) Follicular epithelium of vitellogenic oocytes from control females showing the nuclei distribution of the follicular epithelium by DAPI staining. Scale bar: 100 µm. (**L**) Ovariole of silenced (RNAi^*BicD*^) females showing the nuclei distribution by DAPI staining. Scale bar: 100 µm. (**M**) Follicular epithelium in vitellogenic oocytes from silenced (RNAi^*BicD*^) females showing the nuclei distribution of the follicular epithelium by DAPI staining. Scale bar: 100 µm.

To extend the validation, we further studied the ortholog gene *Rp-BicD* (Additional file 8) by in situ hybridization and parental RNAi. Due to the limitation of the parental RNAi technique to distinguish maternal from zygotic phenotypes, we selected a gene that displayed a maternal, stage-specific expression, during oogenesis and early development of *R. prolixus* and that has not been studied in other insect beyond *D. melanogaster*. The structure of an ovariole is shown in Fig. 5A-C. The expression of *Rp-BicD* is cytoplasmic and the mRNA is detected by the antisense probe in both, the germarium and follicular epithelium of previtellogenic and vitellogenic oocytes, while the sense probe does not show any sign of hybridization (Fig. 5D-F). *Rp-BicD* mRNA is also detected in unfertilized eggs confined in the central region of the egg, with a diffusion of the signal towards the embryo surface (Fig. 5G). In embryos up to the onset of blastoderm stage, *Rp-BicD* mRNA could not be detected (data not shown), in agreement with our qRT-PCR data, although we cannot rule out transcripts below the detection limit of the in situ hybridization technique. Similar to *D. melanogaster*, the only insect species in which *BicD* has been studied, the expression decays just prior to the formation of the cellular blastoderm^102^. The dynamic of *Rp-BicD* expression suggests that the maternal transcript occurs during the initial nuclear cleavages but is degraded before cellularization of the blastoderm^9,103^.

In order to determine the function of *Rp-BicD* we performed parental RNAi. We injected non-fed virgin females (n = 20) with different concentrations (0.5 to 2.5 µg/female) of dsRNA^*BicD*^. As control, we used dsRNA dsRNA^*β-lac*^ (see methods). After feeding and mating, dsRNA^*BicD*^ and dsRNA^*β-lac*^ injected females were evaluated for survival, egg deposition, embryo lethality, and ovary and/or embryonic phenotype (Additional file 9). In our hands, 45% females injected with dsRNA^*BicD*^ did not survived to reach mating and egg deposition. Fertility was assessed in the surviving females (n = 9) by the number of eggs laid, while embryo lethality was studied by incubation of the eggs for the time of embryogenesis to finish (> 14 days). Compared to the control, the silenced females laid less eggs (n = 43). From these, only four corresponding to the group injected with the lowest concentration of dsRNA^*BicD*^ hatched to first-instar larvae (Additional file 9). The freshly laid eggs from both control and silenced females have the characteristic pink color due to the presence of the *Rhodnius* heme-binding protein (RHBP) in the yolk and do not show any visible chorion abnormality (Supplementary Fig. 10). As development proceeds, we monitor the embryogenesis by the coloration of the embryo, which can be observed through the chorion, white and transparent^101^. The control eggs showed the development of embryo pigmentation through the chorion, eventually resulting in hatchlings. The eggs derived from silenced females did not show evidence of pigmentation, suggesting that the development was arrested. The dissection of fixed silenced eggs confirmed the absence of any distinguishable embryonic structure, suggesting that *BicD* might act at very early stages of embryogenesis. DAPI staining of fixed early embryos showed that, different than the control, the nuclei are not observed in the surface, thus, the blastoderm was not formed. This suggests that the lack of *Rp-BicD* might affect a process as early as the embryonic nuclear cleavage. (Fig. 5H-I). The morphology of the ovary analyzed under the dissection microscope and the cellular pattern, oocyte and follicle cells, as judged by nuclear staining, did not show conspicuous differences between the control and silenced females. Therefore, silencing of *Rp*-*BicD* did not alter the normal morphology of the ovaries (Fig. 5J-M). The unaffected pattern of the follicle cells agrees with the normal pattern observed in the chorion in eggs derived from silenced females. The observed phenotype shows some differences with the *BicD* phenotype in *D. melanogaster*. In heterozygous *BicD* females the progression of oogenesis is not affected, but it causes sterility or inviable embryos that consist of a mirror-image duplication of 2–4 posterior segments^104,105^. Homozygous *BicD* females show ovaries with no diploid germ cell nuclei visible in older egg chambers and no oocyte development. The lack of *BicD* affects the zygotic viability, in which null flies die as pupae or young adults^106^. Our data indicate that in *R. prolixus* the function might be affecting the earliest steps of embryogenesis, but not the formation of the egg. Interestingly, in *D. melanogaster*, *BicD* and *Egalitarian* (*Egl*) are part of a complex that transports and localizes mRNAs in the oocyte, crucial for specifying the embryo axes^104,107–109^. *Egl* and *BicD* homologues have been identified in *Caenorhabditis elegans* and mammals, and proposed as a part of an evolutionarily conserved cytoskeletal system for mRNA transport^110,111^. Our qRT-PCR results show similar expression dynamics for *Rp-BicD* and *Rp-egl*, suggesting that this contemporary expression might reflect the conservation of the *BicD*/*Egl* localization machinery in *R. prolixus*, an aspect yet unexplored in this insect.

## Conclusions

Our data provide a framework for functional studies of *R. prolixus* oogenesis and embryogenesis. We identified at least three temporal patterns of gene expression: 1. genes that are maternally expressed and rapidly decreased (*Rp-capu*, *Rp-egh*, *Rp-exu*); 2. genes with transient expression (*Rp-arm*, *Rp-BicD*, *Rp-egl*, *Rp-sqd*, *Rp-pb*, *Rp-stau*); 3. genes with invariant expression (*Rp-cact*, *Rp-dl*, *Rp-pum*). These dynamic suggest temporal roles based on known phenotypes: 1. Early oogenesis (genes that affect germ cell survival or results in atresic follicles): *Rp-ATG-8*^112^; *Rp-cactus*^46^; *Rp-Piwi-2*, *Rp-Piwi-3*, *Rp-Argonauta-3*^48^; 2. Late oogenesis (genes that results in eggs with incomplete yolk load or impaired choriogenesis): *Rp-Bicaudal C*^50^; *ULK/Rp-ATG-1*^113^; *Rp-ATG-3*^114^; *Rp-ATG-6*^115^; 3. Embryonic patterning genes: *Rp-dorsal*^46^; *Rp-Bicaudal D* (this work).

Genome analysis opened an exciting path to study the molecular mechanism involved in *R. prolixus* oogenesis, a model established by the pioneer work of Erwin Huebner. These studies will enrich our knowledge on the evolution of development and, in the case of *R. prolixus,* might contribute to envisage new strategies to control the reproduction of Chagas disease vectors.

## Supplementary Information

Supplementary Information 1.

Supplementary Information 2.

Supplementary Information 3.

Supplementary Information 4.

Supplementary Information 5.

Supplementary Information 6.

Supplementary Information 7.

Supplementary Information 8.

Supplementary Information 9.

Supplementary Information 10.

Supplementary Information 11.
