# Author Correction: Dynamics of maternal gene expression in *Rhodnius prolixus*

**DOI:** 10.1038/s41598-022-23779-5

**Published:** 2022-11-09

**Authors:** Agustina Pascual, Rolando Rivera‑Pomar

**Affiliations:** 1 Centro de BioInvestigaciones (CeBio‑CICBA), Universidad Nacional del Noroeste de la Provincia de Buenos Aires (UNNOBA), Avenida Presidente Frondizi 2650, 2700 Pergamino, Buenos Aires Argentina; 2 Centro de Investigaciones y Transferencias del Noroeste de la Provincia de Buenos Aires (CITNOBA‑CONICET), UNNOBA-UNSAdA, Consejo Nacional de Investigaciones Científicas y Técnicas (CONICET), Buenos Aires, Argentina; 3 Centro Regional de Estudios Genómicos, Facultad de Ciencias Exactas, Universidad Nacional de la Plata, Bvd 120 y 62, 1900 La Plata, Buenos Aires Argentina

Correction to: *Scientific Reports* 10.1038/s41598-022-09874-7, published online 20 April 2022

The original version of this Article contained an error in Figure 4, where the bar graph showing the expression level of the *Rp-pb* gene, was omitted. The original Figure 4 and accompanying legend appear below.

**Figure 4:**
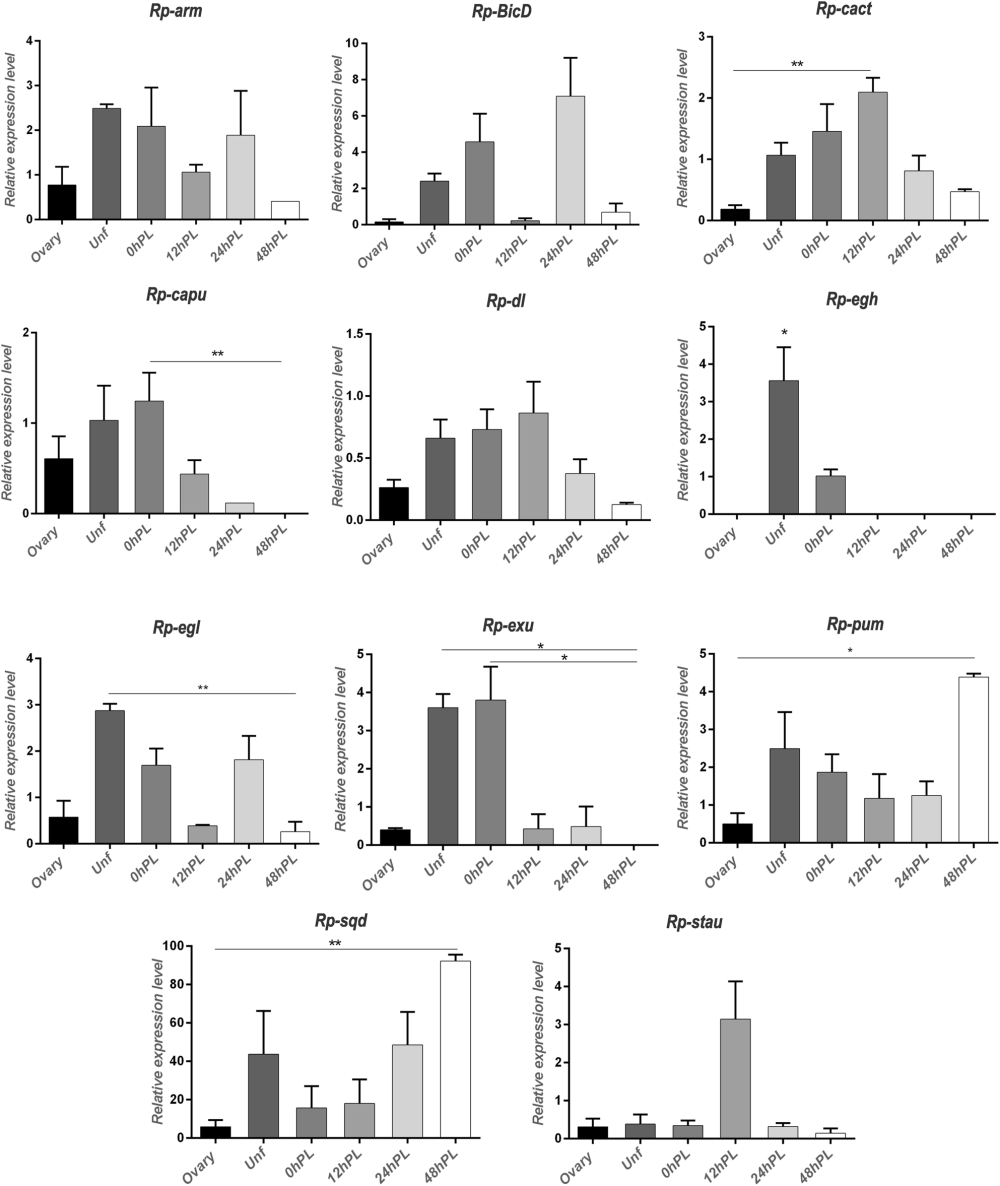
Real-time quantitative PCR of expression of candidate genes in the different stages. X axis: developmental times analyzed. Y axis: expression relative to the reference gene. Values are expressed as mean ± SEM of 3 independent experiments. *Rp-arm*: *Armadillo*; *Rp-BicD: Bicaudal D*; *Rp-cact: Cactus*; *Rp-capu: Cappuccino*; *Rp-dl: Dorsal*; *Rp-egh: Egghead*; *Rp-egl: Egalitarian*; *Rp-exu: Exuperantia*; *Rp-pb: Proboscipedia*; *Rp-pum: Pumilio*; *Rp-sqd: Squid*; *Rp-stau: Staufen*. Graphs were performed using GraphPad Prism 7. * < 0.1; ** < 0.05.

The original Article has been corrected.

