# Supplementary material for: Dynamics of maternal gene expression in Rhodnius prolixus
Source: Sci Rep. 2022 Apr 20;12:6538. doi: 10.1038/s41598-022-09874-7 (PMC9023505; doi:10.1038/s41598-022-09874-7)

Additional file 8

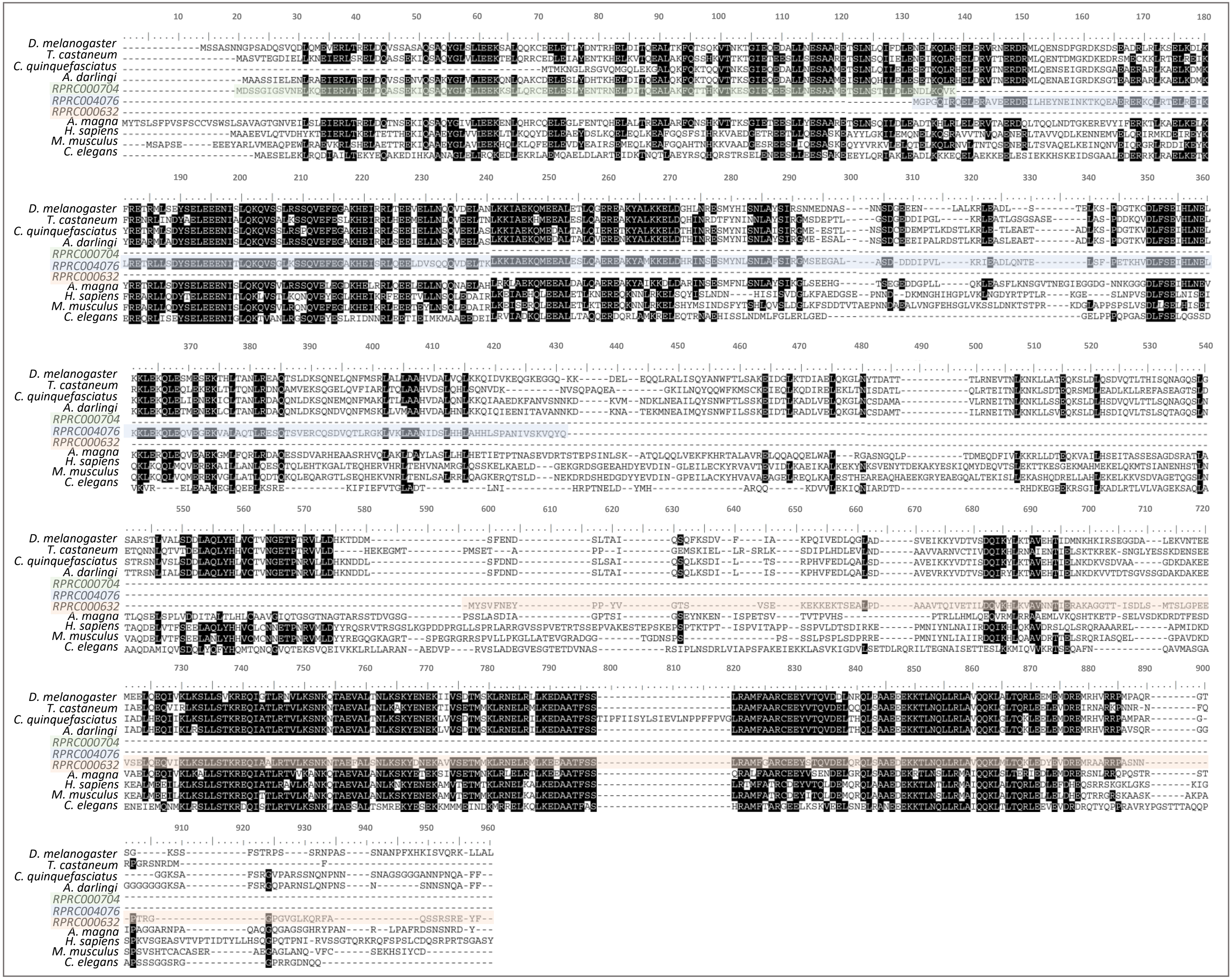

Supplement: Supplementary file 8 — Supplementary Information 8. [file 41598_2022_9874_MOESM8_ESM.pdf]
