# Supplementary figures and images for: Dynamics of maternal gene expression in Rhodnius prolixus
Source: Sci Rep. 2022 Apr 20;12:6538. doi: 10.1038/s41598-022-09874-7 (PMC9023505; doi:10.1038/s41598-022-09874-7)

**Additional file 10**

dsRNA<sup>*β-lac*</sup>

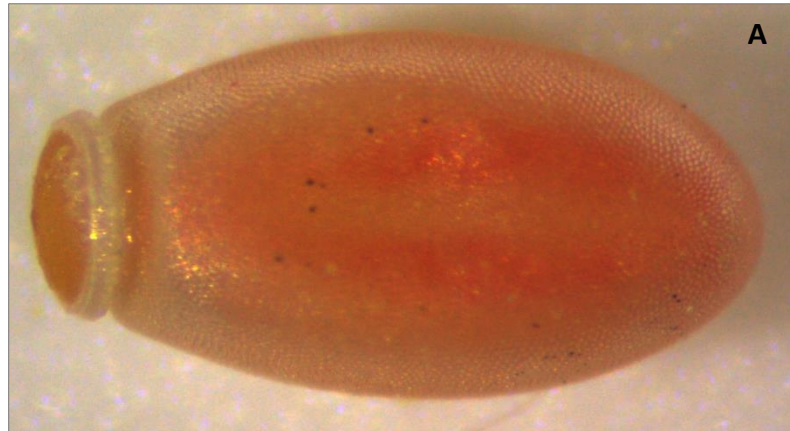

dsRNA<sup>*BicD*</sup>

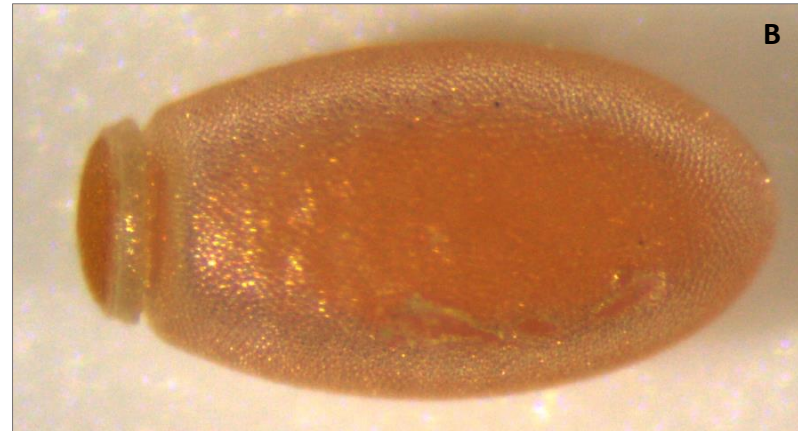

Supplement: Supplementary file 10 — Supplementary Information 10. [file 41598_2022_9874_MOESM10_ESM.pdf]
