# Supplementary material for: Dynamics of maternal gene expression in Rhodnius prolixus
Source: Sci Rep. 2022 Apr 20;12:6538. doi: 10.1038/s41598-022-09874-7 (PMC9023505; doi:10.1038/s41598-022-09874-7)
Supplement: Supplementary file 11 — Supplementary Information 11. [file 41598_2022_9874_MOESM11_ESM.docx]

**Supporting information**

**Additional file 1:** Maternal *D. melanogaster* genes, reported as FlyBase symbol.

**Additional file 2:** Primers used by RT-qPCR assays and *Rp-BicD* experiments.

**Additional file 3:** COG functional category annotation using eggNOG-mapper.

**Additional file 4:** InterproScan results.

**Additional file 5:** GO-term enrichment.

**Additional file 6:** GO-term enrichment form annotated transcripts common to all six developmental stages.

**Additional file 7:** BLASTX from maternal genes.

**Additional file 8:** The predicted sequences were aligned with orthologues from other species with Clustal Ω ^[118](#bookmark)^. Alignment of the protein sequence of *Rp-BicD* with orthologues species, extracted from NCBI sequence database *D. melanogaster* (NP_001260531.1), *Tribolium castaneum* (EFA07458.1), *Culex quinquefasciatus* (EDS37197.1), *Anopheles darlingi* (ETN62092.1), *Daphnia magna* (JAN71328.1), *Homo sapiens* (AAB94805.1), *Mus musculus* (NP_001034268.1), *Caenorhabditis elegans* (CDK13419.1). The amino acid conservation is visualized with black blocks, the amino acid group level conservation with gray blocks.

**Additional file 9:** Summary of Parental RNAi experiment.

**Additional file 10:** Eggs derived from control (A) and silenced (B) females.
